# Validation of Claims-Based Algorithm for Lyme Disease, Massachusetts, USA

**DOI:** 10.3201/eid2909.221931

**Published:** 2023-09

**Authors:** Noelle M. Cocoros, Sheryl A. Kluberg, Sarah J. Willis, Susan Forrow, Bradford D. Gessner, Cameron T. Nutt, Alejandro Cane, Nathan Petrou, Meera Sury, Chanu Rhee, Luis Jodar, Aaron Mendelsohn, Emma R. Hoffman, Robert Jin, John Aucott, Sarah J. Pugh, James H. Stark

**Affiliations:** Harvard Medical School and Harvard Pilgrim Health Care Institute, Boston, Massachusetts, USA (N.M. Cocoros, S.A. Kluberg, S.J. Willis, S. Forrow, C. Rhee, A. Mendelsohn, E.R. Hoffman, R. Jin);; Pfizer Inc., New York, New York, USA (S.J. Willis);; Pfizer Inc., Collegeville, Pennsylvania, USA (B.D. Gessner, A. Cane, L. Jodar, S.J. Pugh, J.H. Stark);; Brigham and Women’s Hospital, Boston (C.T. Nutt, N. Petrou, M. Sury, C. Rhee);; Johns Hopkins School of Medicine, Baltimore, Maryland, USA (J. Aucott)

**Keywords:** Lyme disease, healthcare, healthcare insurance, claims database, algorithm, bacteria, parasites, vector-borne infections, zoonoses, Massachusetts, United States

## Abstract

Compared with notifiable disease surveillance, claims-based algorithms estimate higher Lyme disease incidence, but their accuracy is unknown. We applied a previously developed Lyme disease algorithm (diagnosis code plus antimicrobial drug prescription dispensing within 30 days) to an administrative claims database in Massachusetts, USA, to identify a Lyme disease cohort during July 2000–June 2019. Clinicians reviewed and adjudicated medical charts from a cohort subset by using national surveillance case definitions. We calculated positive predictive values (PPVs). We identified 12,229 Lyme disease episodes in the claims database and reviewed and adjudicated 128 medical charts. The algorithmʼs PPV for confirmed, probable, or suspected cases was 93.8% (95% CI 88.1%–97.3%); the PPV was 66.4% (95% CI 57.5%–74.5%) for confirmed and probable cases only. In a high incidence setting, a claims-based algorithm identified cases with a high PPV, suggesting it can be used to assess Lyme disease burden and supplement traditional surveillance data.

Lyme disease is the most commonly reported vectorborne disease in the United States (*1*) and is an economic burden for patients and society (*2*–*4*). As a notifiable disease, standard Lyme disease case definitions and reporting criteria have identified ≈30,000 cases annually via traditional surveillance (*5*). Several jurisdictions have used alternative methods to approximate Lyme disease incidence, including sampling (*6*), estimation techniques (*7*), and supplementing laboratory-based surveillance data with information from electronic health records (*8*).

To complement traditional surveillance, the Centers for Disease Control and Prevention (CDC) used a commercial health care administrative claims database to estimate Lyme disease incidence in the United States. In 2015, claims-based algorithms were developed for inpatient and outpatient settings; the outpatient algorithm combined diagnosis codes from the International Classification of Diseases, 9th Revision, Clinical Modification (ICD-9-CM), for Lyme disease with dispensing of an antimicrobial drug within 30 days (*9*). That study estimated that ≈329,000 annual cases of Lyme disease occurred during 2005–2010 after applying several correction factors to account for database limitations. The analysis was repeated for cases during 2010–2018 after the addition of diagnosis codes from the International Classification of Diseases, 10th Revision, Clinical Modification (ICD-10-CM), for Lyme disease, estimating that ≈476,000 Lyme disease cases occurred annually during this period (*10*). However, the accuracy of the algorithms is unknown (*11*). We validated this outpatient algorithm by assessing algorithm performance across age groups, healthcare facility type, and periods in a single Lyme disease–endemic state.

## Methods

### Study Population

We used Harvard Pilgrim Health Care (HPHC) administrative claims data to identify the initial Lyme disease cohort in Massachusetts, USA. HPHC is a not-for-profit health insurance company serving >3 million members primarily in the New England region of the United States. HPHC members are approximately half female and half male, and ≈20% of members are >65 years of age. We included HPHC members who were enrolled in medical and pharmacy benefits for >6 months from July 1, 2000, through June 30, 2019, and who were residents of Massachusetts at the time of enrollment.

To validate cases identified in the administrative claims database, we reviewed medical charts for a subset of patients with Lyme disease episodes who received care from any facility that was part of the Mass General Brigham (MGB) healthcare system. We limited chart review to a single healthcare system to simplify accessing medical charts. MGB, the largest provider system in Massachusetts, comprises 16 institutions across the care continuum and has 6,500 physicians. The system includes academic medical centers, specialty and community hospitals, and urgent and community-based care via community health centers that are geographically dispersed across eastern Massachusetts. In 2020, the MGB healthcare system was responsible for ≈20% of inpatient discharges and ≈27% of outpatient revenue in Massachusetts (*12*). We expected the MGB healthcare system to be representative of care delivered across the state.

### Algorithm Criteria and Descriptive Analyses

Lyme disease was defined by >1 diagnosis code (ICD-9-CM code 088.81; ICD-10-CM codes A69.20, A69.21, A69.22, A69.23, and A69.29) and >1 outpatient dispensing of an antimicrobial drug used to treat Lyme disease according to Infectious Diseases Society of America guidelines (*13*). We defined antimicrobial drugs by using the US Food and Drug Administration National Drug Codes for doxycycline, amoxicillin, cefuroxime axetil, azithromycin, penicillin G, ceftriaxone, and cefotaxime; we included oral and nonoral formulations. We required a minimum 7-day supply of the antimicrobial drug and for it to be dispensed within 30 days of the Lyme disease diagnosis. We evaluated the use of doxycycline, amoxicillin, cefuroxime axetil, azithromycin, penicillin G, ceftriaxone, and cefotaxime to treat Lyme disease.

To identify Lyme disease episodes, we required that HPHC members did not have a Lyme disease diagnosis code documented within 180 days before meeting the Lyme disease definition (i.e., if someone had a Lyme disease diagnosis code but no antimicrobial drug dispensed and then had another Lyme disease diagnosis code <180 days later with a qualifying antimicrobial drug dispensed, we did not include the second episode). For members who had multiple Lyme disease episodes, we used recurrence intervals to exclude episodes in which the diagnosis code and antimicrobial drug were likely used for treating Lyme disease–related sequelae from the first infection; we used intervals according to those used by others for ICD-9-CM (*9*) and ICD-10-CM (*10*) codes. During the ICD-9-CM era (before October 1, 2015), the recurrence interval was 365 days. During the ICD-10 era (beginning October 1, 2015), if a member met the algorithm definition with code A69.2 (Lyme disease) or A69.20 (Lyme disease, unspecified) on or after October 1, 2015, the recurrence interval was 180 days, as long as the second Lyme disease case date was in the next calendar year. If the second Lyme disease case date was in the same calendar year, then the second episode was not included. If a member met the algorithm definition with code A69.21 (meningitis), A69.22 (other neurologic disorders), A69.23 (arthritis), or A69.29 (other conditions) on or after October 1, 2015, the recurrence interval was 365 days.

We summarized characteristics of HPHC members with algorithm-defined Lyme disease during the full study period by using descriptive statistics. We examined the frequencies and percentages of patient demographic and clinical characteristics associated with Lyme disease episodes that were available in the administrative claims data. Acute signs and symptoms were rash, fever, chills, fatigue, headache, joint and muscle pain, radiculopathy, and paresthesia, and those were identified by ICD-9-CM and ICD-10-CM diagnosis codes reported within 14 days before or after meeting the Lyme disease algorithm definition (Appendix Table). Musculoskeletal, nervous system, cardiovascular, and ocular manifestations of Lyme disease were examined up to 1 year after Lyme disease diagnosis and were also identified by diagnosis codes (Appendix Table). Among those patients with obtainable MGB medical records that were reviewed and adjudicated, we evaluated demographic and clinical characteristics and summarized acute symptoms and disseminated manifestations by using the same criteria described previously. We also assessed laboratory data captured in the medical records to determine how many cases were laboratory-confirmed.

### Algorithm Validation via Medical Chart Reviews

We had an a priori goal of reviewing 125 medical charts for algorithm validation; we prioritized cases from the ICD-10-CM era and then included ICD-9-CM era episodes to obtain >125 charts. We identified 193 medical charts for persons with HPHC insurance who had evidence of Lyme disease–related care at a facility within the MGB system and who met the algorithm criteria during January 2015–June 2019; we sought medical records for a convenience sample of 171 cases.

Under the supervision of an infectious disease clinical faculty member (C.R.), 3 MGB medical residents (C.T.N., N.P., M.S.) conducted all chart abstraction and adjudication activities. Prior to conducting those activities, they received training from a Lyme disease clinical expert (J.A.). To assess interrater reliability, all 3 clinicians initially abstracted and adjudicated the same 20 medical charts. We calculated a single κ-like statistic that summarized interrater reliability across all clinicians by computing the mean of the weighted κ for each clinician pair (*14*). We divided the remaining charts among the 3 clinicians for single adjudications.

We conducted medical chart reviews assuming that the clinician-determined adjudication was the standard for definitively assigning Lyme disease status according to surveillance case definitions. We developed standardized abstraction and adjudication forms for chart reviews that had definitions consistent with the 2017 Council of State and Territorial Epidemiologists’ Lyme disease case definitions for confirmed, probable, and suspected cases (*15*) (Appendix). Abstracted data from each medical record were evidence of erythema migrans or rash; tick bite or exposure to ticks; signs and symptoms of Lyme disease; cardiovascular, musculoskeletal, or nervous system manifestations of Lyme disease; antimicrobial drugs or other medications used to treat Lyme disease; laboratory tests and results; physician diagnosis of Lyme disease; evidence of persistent signs and symptoms of Lyme disease; and healthcare facility type. Claims-based Lyme disease cases were adjudicated, and we classified each case as confirmed, probable, suspected, or not a Lyme disease case (Table 1).

**Table 1:** Case classification, definitions, and instructions used by clinician adjudicators for chart review in study of validation of claims-based algorithm for Lyme disease, Massachusetts, USA*

Classification	Definitions†
Confirmed	Erythema migrans with known exposure in a high-incidence state (e.g., Massachusetts), erythema migrans with known exposure in a low-incidence state and laboratory-confirmed Lyme disease, or >1 late manifestation of Lyme disease and laboratory-confirmed Lyme disease
Probable	Diagnosis of Lyme disease in clinical notes and laboratory-confirmed Lyme disease but no evidence of erythema migrans and no eligible late manifestations of disease
Suspected	Diagnosis of Lyme disease in clinical notes and antimicrobial drugs ordered by healthcare provider to treat Lyme disease but no laboratory confirmation, no evidence of erythema migrans, and no eligible late manifestations of Lyme disease; or erythema migrans with no known exposure, no laboratory confirmation, and no eligible late manifestations of Lyme disease

*Definitions were based on the 2017 Council of State and Territorial Epidemiologists case definitions (*15*). †Laboratory-confirmed Lyme disease was indicated by positive Lyme cultures, PCR, or 2-tiered tests. For a positive 2-tiered test, if the patient experienced signs or symptoms for <30 d before a positive or equivocal enzyme immunoassay or immunofluorescence assay, they must have a positive IgG or IgM Western blot result; if the patient has experienced signs or symptoms for >30 d before a positive or equivocal enzyme immunoassay or immunofluorescence assay, they must have a positive IgG Western blot result (a positive IgM Western blot result does not confirm Lyme disease in this scenario). Late manifestations of Lyme disease include musculoskeletal involvement defined as inflammatory arthritis or recurrent and brief attacks of swelling in >1 joint that lasts for several weeks or months; nervous system involvement defined as lymphocytic meningitis, cranial neuritis, radiculoneuropathy, or encephalomyelitis (headache, fatigue, paresthesia, or mildly stiff neck alone did not meet criteria for neurologic involvement); or cardiovascular involvement defined as acute onset of high-grade atrioventricular conduction defects that resolve in days to weeks, such as complete heart block, third degree heart block, or high-grade atrioventricular block (palpitations, bradycardia, bundle branch block, or myocarditis alone did not meet criteria for cardiovascular involvement).

We calculated positive predictive values (PPV) for claims-based Lyme disease cases adjudicated as confirmed, probable, or suspected and PPV values for confirmed or probable cases only. We calculated PPVs according to age group, healthcare facility type, period, and patients with Lyme disease–related laboratory tests to determine how performance varied across those subgroups. We used the Clopper-Pearson method to calculate 95% CIs for all PPVs (*16*). The study was approved by the Harvard Pilgrim Health Care Institutional Review Board.

## Results

### Claims Data

From July 1, 2000, through June 30, 2019, by using the Lyme disease claims-based algorithm, we identified 12,229 Lyme disease episodes among 11,823 HPHC members who lived in Massachusetts; a total of 11,452 members had 1 Lyme disease episode, 339 had 2 episodes, and 32 had 3 or 4 qualifying episodes. Most (77.7%) episodes were identified during the ICD-9-CM era; the only applicable code was 088.81, Lyme disease. During the ICD-10-CM era, the most common cohort-defining diagnosis code was A69.20, Lyme disease unspecified (93.0%); 4.9% were identified as A69.23, arthritis due to Lyme disease; 1.4% as A69.29, other conditions associated with Lyme disease; and <1% as A69.22, other neurologic disorders in Lyme disease, or A69.21, meningitis due to Lyme disease.

We analyzed demographic and clinical characteristics of patients with Lyme disease episodes according to claims data for the overall cohort (n = 12,229) and the subset included in the chart review (n = 128) (Table 2). Most Lyme disease episodes occurred among adults >18 years of age, including 71.4% in the overall cohort (median age 42 years, interquartile range 15–55 years; 45.9% were 40–64 years of age) and 80.5% in the chart review (median age 48 years, interquartile range 29–60 years; 48.4% were 40–64 years of age). Male patients comprised 49.2% of reviewed charts and 54.6% of all Lyme disease episodes.

**Table 2:** Demographic and clinical characteristics of Harvard Pilgrim Health Care members who met criteria for a validation study of a claims-based algorithm for Lyme disease during July 2000–June 2019 in Massachusetts, USA*

Characteristics	Total Lyme disease episodes	Lyme disease episodes included in chart review
Total no. cases	12,229	128
Age groups, y		
Pediatric, <18	3,494 (28.6)	25 (19.5)
Adult, >18	8,735 (71.4)	103 (80.5)
<1	8 (0.1)	0
1–4	775 (6.3)	8 (6.3)
5–14	2,271 (18.6)	16 (12.5)
15–24	1,208 (9.9)	7 (5.5)
25–39	1,500 (12.3)	16 (12.5)
40–64	5,609 (45.9)	62 (48.4)
>65	858 (7.0)	19 (14.8)
Median age, y (IQR)	42 (15–55)	48 (29–60)
Sex		
M	6,675 (54.6)	63 (49.2)
F	5,554 (45.4)	65 (50.8)
Antimicrobial drug		
Doxycycline	8,110 (66.3)	107 (83.6)
Amoxicillin	3,594 (29.4)	18 (14.1)
Cefuroxime axetil	341 (2.8)	2 (1.6)
Azithromycin	177 (1.5)	1 (0.8)
Penicillin G	7 (0.1)	0
Acute signs and symptoms†		
No signs or symptoms	6,931 (56.7)	80 (62.5)
Joint pain	1,681 (13.7)	12 (9.4)
Rash‡	1,644 (13.4)	20 (15.6)
Fatigue	1,518 (12.4)	10 (7.8)
Fever	1,091 (8.9)	6 (4.7)
Headache	999 (8.2)	7 (5.5)
Myalgia	654 (5.3)	10 (7.8)
Disseminated manifestations§		
Nervous system	904 (7.4)	9 (7.0)
Musculoskeletal	326 (2.7)	10 (7.8)
Ocular	257 (2.1)	3 (2.3)
Cardiovascular	37 (0.3)	0

*Values are no. (%) except as indicated. All data are from the Harvard Pilgrim Health Care administrative claims database. The 128 Lyme disease episodes included in the chart reviews were also included in the total Lyme disease episode data. IQR, interquartile range. †Rash, fever, chills, fatigue, headache, joint pain, neck pain or stiff neck, radiculopathy, myalgia, and paresthesia were derived from diagnosis codes from the International Classification of Diseases, 9th Revision, Clinical Modification, and International Classification of Diseases, 10th Revision, Clinical Modification, documented up to 14 d before or after the member met the claims-based definition of Lyme disease. ‡Upon medical record review, 62 of 128 (48.4%) cases had evidence of erythema migrans. §Nervous system, musculoskeletal, ocular, and cardiovascular manifestations were derived from diagnosis codes from the International Classification of Diseases, 9th Revision, Clinical Modification, and International Classification of Diseases, 10th Revision, Clinical Modification, documented up to 365 d after the member met the claims-based definition of Lyme disease. A patient can have both disseminated manifestations and acute signs and symptoms.

Of the total Lyme disease episodes, 66.3% were associated with dispensation of a >7-day supply of doxycycline, 29.4% with amoxicillin, and 4.3% with cefuroxime acetyl, azithromycin, or penicillin G. Some cases (2%) were treated with >1 antimicrobial drugs. No cases were treated with ceftriaxone or cefotaxime. Within the subset included in the chart review, the pattern was similar, although more patients (83.6%) were treated with doxycycline.

For the overall Lyme disease cohort, during the 14 days before and after the Lyme disease case date, 56.7% of cases did not have any diagnosis codes recorded in claims data that were indicative of acute signs or symptoms; 13.7% of cases had diagnosis codes for joint pain, 13.4% for rash, and 12.4% for fatigue. During the 365 days after the Lyme disease case date, 7.4% of cases had a diagnosis code indicative of a nervous system manifestation, such as Bell’s palsy, meningitis, or radiculopathy. Musculoskeletal (2.7%), ocular (2.1%), or cardiovascular (0.3%) manifestations occurred within 365 days of the Lyme disease case date, according to diagnosis codes. Those findings were generally similar among patients included in chart reviews.

### Algorithm Validation via Chart Review

Of the 128 (75%) obtainable medical records that we reviewed and adjudicated, 80.5% were for cases that occurred during the ICD-10 era. The overall interrater reliability for the 20 charts reviewed by all 3 clinician adjudicators yielded a mean weighted κ of 0.94.

Overall, we adjudicated 120 of 128 reviewed charts as confirmed, probable, or suspected cases. The distribution of those 120 cases followed the expected seasonality of Lyme disease in Massachusetts; the peak was observed in July. Of the 18.8% of cases that were laboratory-confirmed (defined by positive Lyme disease culture, PCR, or standard 2-tiered tests), all were adjudicated as confirmed or probable cases. A clinical diagnosis of Lyme disease was indicated in 55.5% of charts, defined as erythema migrans or Lyme disease–associated carditis, neuroborreliosis, meningitis, or arthritis in the healthcare provider’s clinical notes. Upon chart review, erythema migrans was reported for 48% of patients (98.4% of whom were adjudicated as confirmed cases), which was substantially higher than the 15.6% of patients with evidence of rash via claims data alone. Similar to observations for claims data alone, reports of disseminated Lyme disease manifestations were uncommon upon chart review. Musculoskeletal involvement was found in 6.3%, nervous system involvement in 2.3%, cardiovascular involvement in <1%, and ocular involvement in 0% of cases; 75% (n = 9) of patients with a disseminated manifestation were adjudicated as confirmed cases.

For reviewed charts, we calculated PPVs for the algorithm overall and according to select characteristics (Table 3). Most (74.2%) charts were from patients seen in a primary care setting. The overall PPV of the algorithm for cases identified as confirmed, probable, or suspected was 93.8% (95% CI 88.1%–97.3%). When limited to confirmed or probable cases only, the PPV was 66.4% (95% CI 57.5%–74.5%). The PPV for confirmed, probable, or suspected cases was 100% (n = 25) for pediatric patients, compared with 92.2% (n = 103) for adult patients. PPVs for confirmed, probable, and suspected cases were 92.0% for those identified during the ICD-9 era and 92.4% for those identified during the ICD-10 era. When including only confirmed and probable cases, the PPV was 76.0% for the ICD-9 era and 64.1% for the ICD-10 era.

**Table 3:** Numbers of reviewed charts and positive predictive values according to case definitions and other factors during January 2015–June 2019 in study of validation of claims-based algorithm for Lyme disease, Massachusetts, USA*

Factors	Reviewed charts	Confirmed	Probable	Suspected	Not LD	% PPV (95% CI)†	% PPV (95% CI)‡
Overall	128	70	15	35	8	93.8 (88.1–97.3)	66.4 (57.5–74.5)
Age group, y							
Pediatric, <18	25 (19.5%)	19	3	3	0	100 (86.3–100)	88 (68.8–97.5)
Adults, >18	103 (80.5%)	51	12	32	8	92.2 (85.3–96.6)	61.2 (51.1–70.6)
ICD era§							
ICD-9-CM	25 (19.5%)	16	3	4	2	92.0 (74.0–99.0)	76.0 (54.9–90.6)
ICD-10-CM	103 (80.5%)	54	12	31	6	94.2 (87.8–97.8)	64.1 (54.0–73.3)
Healthcare facility type							
Primary care	95 (74.2%)	51	14	26	4	95.8 (89.6–98.8)	68.4 (58.1–77.6)
Urgent care	17 (13.3%)	12	1	4	0	100 (80.5–100)	76.5 (50.1–93.2)
Other¶	16 (12.5%)	7	0	5	4	75.0 (47.6–92.7)	31.3 (11.0–58.7)
Laboratory tests#	68 (53.1%)	27	15	21	5	92.7 (83.7–97.6)	61.8 (49.2–73.3)

*Values are no. (%) except as indicated. Lyme disease case definitions are confirmed, probable, suspected, and not Lyme disease. ICD, International Classification of Diseases; ICD-9, ICD, 9th Revision, Clinical Modification; ICD-10, ICD, 10th Revision, Clinical Modification; LD, Lyme disease; PPV, positive predictive value. †Confirmed, probable, or suspected cases. ‡Confirmed or probable cases only. §Case dates were January 2015–September 2015 for ICD-9 and October 2015–June 2019 for ICD-10. ¶Includes specialist practice (n = 5), emergency department (n = 3), telephone encounter (n = 3), and unknown facility type (n = 5). #Laboratory testing performed (regardless of result).

Among the 8 patients who did not have Lyme disease upon adjudication, none had erythema migrans, and 1 patient had a nonspecific rash. Only 1 patient had a documented tick bite. One patient’s chart indicated *Borrelia miyamotoi* infection and another noted a suspected *B. miyamotoi* infection. Among 5 patients who had a Lyme disease test, 4 had negative results documented.

## Discussion

We report high PPVs for a claims-based algorithm previously used by the CDC to estimate the incidence of Lyme disease in the United States, using claims data and medical record information from sources in Massachusetts. The PPV for cases adjudicated as confirmed, probable, or suspected (according to surveillance case definitions) was 93.8%; PPV was 66.4% when limited to only confirmed or probable. Our results provide support for previous studies (*4*,*9*,*10*,*17*,*18*) and future research aimed at using claims-based algorithms to estimate the total burden of Lyme disease.

Algorithm performance varied depending on the inclusion of suspected cases in PPV calculations. The surveillance definition for a suspected case captures persons treated presumptively and those who do not have true Lyme disease as well as those who, for example, have poor recall of a tick bite (and, therefore, no known exposure) or whose erythema migrans resolves before a scheduled medical encounter. Because all suspected cases were treated, they represent a burden on the healthcare system.

The PPV also varied according to the ICD coding era. The ICD-9 era had a higher PPV (76.0% [95% CI 54.9%–90.6%]) than did the ICD-10 era (64.1% [95% CI 54.0%–73.3%]) when restricted to only confirmed and probable cases; 16% of charts reviewed from the ICD-9 era were adjudicated as suspected cases, compared with 30% from the ICD-10 era. The difference in adjudication percentages could be explained by increased awareness of Lyme disease in recent years leading to more presumptive treatment and diagnosis. Of note, most (81%) of the charts reviewed were diagnosed in the ICD-10 era and yielded a narrower CI.

We showed that a low percentage of Lyme disease episodes in both the claims data and chart review subset had evidence of disseminated disease (neurologic, musculoskeletal, and cardiac systems). Some variation existed according to data source; musculoskeletal involvement was the most prevalent (6% of cases) disseminated manifestation identified in the chart review subset, whereas nervous system involvement within 1 year was most common (7% of cases) in the claims-based cohort. Another study also reported low prevalence of disseminated Lyme disease in claims data using the same algorithm (*19*). Overall, that study found that 6% of Lyme disease episodes had disseminated disease within 30 days of diagnosis; arthritis was the most common manifestation at 3%, followed by facial palsy at 2%. Those findings contrast with surveillance reports indicating 27.5% of patients with confirmed Lyme disease had arthritis, 1.5% had carditis, and 12.5% had a neurologic manifestation (*1*) and another report indicating 43.9% of cases reported via laboratory-based surveillance had evidence of disseminated Lyme disease (*20*). This discrepancy might be because of lack of capture of those conditions in claims data or a lack of ascertainment of disseminated disease with this algorithm, which requires a Lyme disease diagnosis code. Alternatively, estimates of disseminated manifestations in surveillance data might be overestimates because of reporting bias. Previous claims data–based studies have found that >50% of Lyme disease patients did not have a Lyme disease–specific diagnosis code (*9*,*21*). Future studies should aim to elucidate this discrepancy by validating other case-identifying algorithms. Another explanation might be that the algorithm required data on outpatient dispensing of a 7-day antimicrobial drug supply; we did not include procedure codes for treatment with intravenous antimicrobial drugs. Therefore, the algorithm might have underperformed for identifying nervous system disease because treatment of those manifestations includes intravenous antimicrobial drugs. 

We validated the claims-based algorithm to support its use in retrospectively estimating Lyme disease incidence, but claims data can be used for routine ongoing surveillance if data lags are anticipated and understood. The timeliness of settled (closed) claims data varies according to care settings and specific data elements. For example, outpatient drug dispensing data are generally available and complete within several weeks of service, whereas hospital-based claims data can take months to be near-complete.

The first limitation of our study is that we obtained 128 (75%) of 171 charts that were sought for our analysis. Although the number is slightly higher than for other studies that identified charts from claims data for review (*22*–*24*), whether the charts that were unobtainable were more or less likely to contain a Lyme disease diagnosis is unknown. Charts were often unobtainable because the electronic medical records lacked information on the encounter of interest. Second, we validated the algorithm in a Lyme disease–endemic state, and the algorithm might not perform similarly in nonendemic states because of differences in physician awareness and Lyme disease testing, treatment, and coding practices. One study validated a claims-based algorithm for outpatient Lyme disease in Tennessee, a non–Lyme disease–endemic state, and reported a PPV of 5%. However, that study used a different algorithm, which was defined by >3 occurrences of the ICD-9 diagnosis code for Lyme disease (*25*). Future studies should consider validating the algorithm developed by CDC in a non–Lyme disease–endemic state. Third, we were unable to assess the sensitivity or specificity of the algorithm given our study design. Fourth, the chart reviews were conducted within 1 Massachusetts healthcare system, albeit a large system with many different clinical practices and sites. Any claims-based algorithm will perform differently according to testing, treatment, and coding practices, which might vary by clinical practice and system. However, the algorithm we used was not highly specialized, and we hypothesize that its performance would be similar in other Lyme disease–endemic regions. Finally, diagnosis codes for symptoms are generally undercaptured in administrative claims data. Therefore, we might have underestimated the frequency of acute signs and symptoms of Lyme disease in our claims-based analysis and, perhaps, the frequency of late manifestations of Lyme disease as well.

In conclusion, we found that a claims-based algorithm defined by documentation of a Lyme disease diagnosis cods and dispensation of an outpatient antimicrobial drug had a high PPV upon chart validation. Our analysis bolsters previous claims-based estimates of Lyme disease, indicating a substantial burden of medically attended Lyme disease in high-incidence states. Our findings suggest that claims data can be used to estimate Lyme disease incidence by state or nationally. More accurate estimates of Lyme disease incidence can inform decisions related to prevention, both clinically and from a policy perspective. 

Appendix Additional information for validation of claims-based algorithm for Lyme disease, Massachusetts, USA.
