# Supplementary material for: Validation of Claims-Based Algorithm for Lyme Disease, Massachusetts, USA
Source: Emerg Infect Dis. 2023 Sep;29(9):1772–9. doi: 10.3201/eid2909.221931 (PMC10461665; doi:10.3201/eid2909.221931)
Supplement: Appendix — Additional information for validation of claims-based algorithm for Lyme disease, Massachusetts, USA. [file 22-1931-Techapp-s1.pdf]

Article DOI: https://doi.org/10.3201/eid2909.221931

EID cannot ensure accessibility for supplementary materials supplied by authors. Readers who have difficulty accessing supplementary content should contact the authors for assistance.

Validation of Claims-Based Algorithm for Lyme Disease, Massachusetts, USA

Appendix

Appendix Table. Diagnosis codes for signs, symptoms, and manifestations of Lyme disease*

Code Type	Code	Description
Signs and symptoms		
ICD-9-CM	782.1	Rash and other nonspecific skin eruption
ICD-10-CM	R21	Rash and other nonspecific skin eruption
ICD-9-CM	780.60	Fever, unspecified
ICD-10-CM	R50.9	Fever, unspecified
ICD-9-CM	780.64	Chills without fever
ICD-10-CM	R68.83	Chills without fever
ICD-9-CM	780.79	Other malaise and fatigue
ICD-10-CM	G93.3	Post-viral fatigue syndrome
ICD-10-CM	R53.1	Weakness
ICD-10-CM	R53.81	Other malaise
ICD-10-CM	R53.83	Other fatigue
ICD-9-CM	784.0	Headache
ICD-10-CM	R51	Headache
ICD-9-CM	719.XX	Pain in joint
ICD-10-CM	M25.5–M25.5XX	Pain in joint
ICD-9-CM	723.1	Cervicalgia
ICD-10-CM	M54.2	Cervicalgia
ICD-9-CM	729.1	Myalgia and myositis, unspecified
ICD-10-CM	M79.1X	Myalgia
ICD-9-CM	782.0	Disturbance of skin sensation
ICD-10-CM	R20–R20.X	Disturbance of skin sensation
ICD-9-CM	723.4	Radicular syndrome of the upper extremities
ICD-9-CM	724.4	Radicular syndrome of the lower extremities
ICD-10-CM	M54.1–M54.1X	Radiculopathy
Musculoskeletal manifestations		
ICD-10-CM	A69.23	Arthritis from Lyme disease
ICD-9-CM	711.80–711.89	Arthropathy associated with other infections and parasitic diseases
ICD-10-CM	M01.X0–M01.X9	Arthropathy associated with other infections and parasitic diseases (arthritis)
Nervous system manifestations		
ICD-10-CM	A69.21	Meningitis from Lyme disease
ICD-10-CM	A69.22	Other neurologic disorders in Lyme disease
ICD-9-CM	320.7	Meningitis
ICD-10-CM	G01	Meningitis
ICD-9-CM	351.0	Bell’s palsy
ICD-10-CM	G51.0	Bell’s palsy
ICD-9-CM	352.6	Multiple cranial nerve palsies
ICD-9-CM	352.9	Unspecified disorder of cranial nerves
ICD-10-CM	G52.7	Disorders of multiple cranial nerves
ICD-10-CM	G52.8	Disorders of other specified cranial nerves
ICD-10-CM	G52.9	Cranial nerve disorder, unspecified
ICD-9-CM	723.4	Radicular syndrome of the upper extremities
ICD-9-CM	724.4	Radicular syndrome of the lower extremities
ICD-10-CM	M54.1–M54.1X	Radiculopathy
ICD-9-CM	323.9	Unspecified causes of encephalitis, myelitis, and encephalomyelitis
ICD-10-CM	G04.90	Encephalitis and encephalomyelitis, unspecified
Cardiovascular manifestations		
ICD-9-CM	422.0	Myocarditis
ICD-10-CM	I41	Myocarditis
ICD-9-CM	426.0	A-V block, complete

Code Type	Code	Description
ICD-9-CM	426.12	A-V block, second degree Mobitz II
ICD-9-CM	426.13	A-V block, second degree, Mobitz I – Wenckebach
ICD-10-CM	I44.2	A-V block, complete
ICD-10-CM	I44.1	A-V block, second degree
Ocular manifestations		
ICD-9-CM	372.0–372.03	Acute conjunctivitis
ICD-9-CM	372.3, 372.30	Other and unspecified acute conjunctivitis
ICD-10-CM	H10.0, H10.02–H10.029	Mucopurulent conjunctivitis
ICD-10-CM	H10.01–H10.019	Acute follicular conjunctivitis
ICD-10-CM	H10.3–H10.33	Unspecified acute conjunctivitis
ICD-9-CM	370.5, 3705.0, 370.52	Interstitial keratitis
ICD-10-CM	H16.3–H16.329	Interstitial and deep keratitis
ICD-9-CM	360.12	Panuveitis
ICD-10-CM	H44.11–H44.119	Panuveitis
ICD-9-CM	377.31	Optic papillitis
ICD-10-CM	H46.0–H46.03	Optic papillitis
ICD-9-CM	379.0, 379.09	Scleritis and episcleritis
ICD-10-CM	H15.1–H15.109	Episcleritits

*ICD-9-CM, International Classification of Diseases, 9th Revision, Clinical Modification; ICD-10-CM, International Classification of Diseases, 10th Revision, Clinical Modification.

Chart Abstraction Form

Case Identification Number for project:________________
Date patient met claims-based algorithm definition: ___________________

Section 1. Lyme disease components. For all questions in section 1, look for documentation in the medical record up to one month before and up to one month after the date the patient met the claims-based algorithm definition of Lyme disease.

1. Does patient have ***erythema migrans*** (round or oval single well circumscribed lesion, >5cm in diameter) reported by a health care provider in clinical notes, assessment/plan, and/or physical exam?
   Yes/No Estimated date rash began:___________________
   Date of clinical note: _________________________
2. Does patient have documentation of a ***rash*** (details of rash and size not reported) reported by a health care provider in clinical notes, assessment/plan, and/or physical exam?
   Yes/No Estimated date rash began:___________________
   Date of clinical note: ________________________
3. Does patient have documentation of a ***tick bite*** reported by a health care provider in clinical notes, assessment/plan, and/or physical exam?
   Yes/No Estimated date of tick bite: ___________________
   State in which tick bite occurred: ___________________
4. Does patient have documentation of ***exposure to a wooded or grassy area*** reported by a health care provider in clinical notes, assessment/plan, and/or physical exam?
   Yes/No Estimated date of exposure: ___________________
   State in which exposure occurred: ___________________
5. Does patient have ***clinical diagnosis of Lyme disease*** in the health care provider's clinical notes? This includes erythema migrans, Lyme carditis, Lyme neuroborreliosis, Lyme meningitis, and Lyme arthritis.
   Yes/No Date of earliest diagnosis:___________________
   If yes, please indicate which of the following diagnoses were made:

Erythema migrans	Yes	No
Lyme carditis	Yes	No
Lyme neuroborreliosis	Yes	No
Lyme meningitis	Yes	No
Lyme arthritis	Yes	No

6. Does the patient have documentation of the following signs and symptoms in health care provider's clinical notes (includes the history from patient, assessment/plan, and physical exam)?

Signs and symptoms	Earliest date of onset	Symptom present for more than 4 weeks

Fever reported or documented (temperature >100.4°F or 38°C)	Yes/No	__________________	Yes/No/Unknown
Chills	Yes/No	__________________	Yes/No/Unknown
Fatigue	Yes/No	__________________	Yes/No/Unknown
Headache	Yes/No	__________________	Yes/No/Unknown
Stiff neck or neck pain	Yes/No	__________________	Yes/No/Unknown
Arthralgia (joint pain)	Yes/No	__________________	Yes/No/Unknown
Myalgia (muscle soreness or pain)	Yes/No	__________________	Yes/No/Unknown
Swollen lymph nodes	Yes/No	__________________	Yes/No/Unknown
Radiculopathy ("pinched nerve" resulting in pain, weakness, or numbness)	Yes/No	__________________	Yes/No/Unknown
Paresthesia (tingling or pricking sensation on skin)	Yes/No	__________________	Yes/No/Unknown
Problems with cognition or memory	Yes/No	__________________	Yes/No/Unknown

Disseminated signs		Earliest date of onset
Inflammatory arthritis (swollen joint with synovitis, typically knee)	Yes/No	__________________
Recurrent or brief attacks of swelling in one or more joints last weeks or months	Yes/No	__________________
Lymphocytic meningitis - inflammation of membranes covering the brain and spinal cord which can result in headache, neck stiffness, nausea, vomiting, light sensitivity or fever<br>Cerebral spinal fluid will show white blood cell count ≥5 and cell count differential showing a majority of lymphocytes	Yes/No	__________________
Cranial neuritis - particularly seventh nerve (Bell's) palsy or paralysis of facial muscles. This can result in trouble closing eyes, uneven smile, or drooling. Any of the 12 cranial nerves may be involved especially the oculomotor nerves. May be labeled as a "cranial nerve" palsy or deficit	Yes/No	__________________

Radiculoneuropathy - inflammation of the roots of the spinal nerve which can result in numbness or tingling and increased sensitivity to pain in the arms, legs, chest, or face	Yes/No	__________________
Encephalomyelitis - inflammation of the brain tissue which may result in sleepiness, abnormal mood swings, confusion, cognitive changes, personality or behavior changes, hallucinations, or seizures.	Yes/No	__________________
High-grade atrioventricular conduction defects - may be labeled as "complete heart block" or "third degree heart block" or "high-grade AV block"	Yes/No	__________________
Second degree AV block/heart block	Yes/No	__________________
First degree AV block/heart block	Yes/No	__________________

7. Location of medical care for first Lyme disease encounter up to one month before or after the patient meets the claims-based algorithm definition of Lyme disease?
   Primary care practice Yes/No
   Emergency department Yes/No
   Urgent care facility Yes/No
   Hospital, outpatient Yes/No
   Hospital, inpatient Yes/No
   Specialist practice Yes/No
   Telephone Yes/No
8. Does the patient have Lyme laboratory results up to one month before or after they met the claims-based algorithm definition of Lyme disease?
   Lyme culture Positive/Negative/Equivocal Order date: _____________
   Result date: _____________
   Lyme EIA or IFA Positive/Negative/Equivocal Order date: _____________
   Result date: _____________
   Lyme IgM western blot Positive/Negative/Equivocal Order date: _____________
   Result date: _____________
   If two of the following three bands are present the IgM Western Blot is positive (−24kDa, −39kDa, −41kDa)
   Lyme IgG western blot Positive/Negative/Equivocal Order date: _____________
   Result date: _____________
   If five of the following ten bands are present the IgG Western Blot is positive (−18kDa, −21kDa, −28kDa, −30kDa, −39kDa, −41kDa, −58kDa, −66kDa, −93kDa)
   Lyme PCR, blood specimen Positive/Negative/Equivocal Order date: _____________
   Result date: _____________
   Lyme PCR, synovial fluid Positive/Negative/Equivocal Order date: _____________
   Result date: _____________
   Lyme PCR, CSF Positive/Negative/Equivocal Order date: _____________
   Result date: _____________

Provide results for all Lyme-related laboratory tests ordered up to one month before and one month after the date the patient met the claims-based algorithm definition of Lyme disease, even if the results are not available until more than one month after.

9. Was the patient prescribed antibiotics up to one month before or after the date they met the claims-based algorithm definition of Lyme disease?

Doxycycline Yes/No
Date of order: _______
Number of days of treatment: _______
Reason for antibiotic (from clinical notes): __________________

Amoxicillin Yes/No
Date of order: _______
Number of days of treatment: _______
Reason for antibiotic (from clinical notes): __________________

Amoxicillin Clavulanate (Augmentin)
Yes/No
Date of order: _______
Number of days of treatment: _______
Reason for antibiotic (from clinical notes): __________________

Cefuroxime axetil Yes/No
Date of order: _______
Number of days of treatment: _______
Reason for antibiotic (from clinical notes): __________________

Azithromycin Yes/No
Date of order: _______
Number of days of treatment: _______
Reason for antibiotic (from clinical notes): __________________

Ceftriaxone Yes/No
Date of order: _______
Number of days of treatment: _______
Reason for antibiotic (from clinical notes): __________________

Tetracycline Yes/No
Date of order: _______
Number of days of treatment: _______
Reason for antibiotic (from clinical notes): __________________

Other antibiotic Name of antibiotic: _______________________
Date of order: __________
Number of days of treatment: _______
Reason for antibiotic (from clinical notes): __________________

Section 2. Persistent Symptoms

1. Does the patient have documentation of the following signs and symptoms in health care provider's clinical notes (includes the history from patient, assessment/plan, and physical exam) between six to twelve months after the date they met the claims-based definition of Lyme disease ?

<u>Signs and symptoms</u>

Fatigue Yes/No

Headache	Yes/No
Stiff neck or neck pain	Yes/No
Arthralgia (joint pain)	Yes/No
Myalgia (muscle soreness or pain)	Yes/No
Radiculopathy ("pinched nerve" resulting in pain, weakness, or numbness)	Yes/No
Problems with cognition or memory	Yes/No

2. Was the patient prescribed antibiotics (Doxycycline, Amoxicillin, Amoxicillin Clavulanate, Cefuroxime acetyl, Azithromycin, Ceftriaxone, or Tetracycline) between six and twelve months after the date they met the claims-based definition of Lyme disease? Yes/No
   Name of antibiotic: ______________________
Date of order: __________
   Number of days of treatment: _______
   Reason for antibiotic (from clinical notes): _________________

Section 3. Brief synopsis of case (to be completed by chart reviewer):

____
____
____
____

Chart Adjudication Form

Case Identification Number for project:_______________

Section 1. Confirmed Lyme disease

1. Erythema migrans with known exposure in a high incidence state (e.g. Massachusetts)
   OR
2. Erythema migrans with known exposure in a low incidence state and laboratory-confirmed Lyme disease*
   OR
3. At least one late manifestation of Lyme disease** and laboratory-confirmed Lyme disease*

   Does the case meet the definition of confirmed Lyme disease? Yes/No/Uncertain

Section 2. Probable Lyme Disease

1. Diagnosis of Lyme disease in clinical notes and laboratory-confirmed Lyme disease* but no evidence of erythema migrans and no eligible late manifestations of disease

Interpretation: Probable cases include patients who report a tick bite or patients who report exposure to wooded/grassy areas but no known tick bite, have symptoms consistent with Lyme disease *(fever, chills, headache, stiff neck/neck pain, arthralgia, myalgia, swollen lymph nodes, radiculopathy, paresthesia, or problems with cognition or memory), and have laboratory-confirmed Lyme disease. These patients do not have a rash consistent with erythema migrans and do not have late manifestations of disease.*

Does the case meet the probable Lyme disease criteria? Yes/No/Uncertain

Section 3. Suspect Lyme Disease

1. Diagnosis of Lyme disease in clinical notes and antibiotics ordered by health care provider to treat Lyme disease but no laboratory confirmation, no evidence of erythema migrans, and no eligible late manifestations of Lyme disease
2. Erythema migrans with no known exposure, no laboratory confirmation, and no eligible late manifestations of Lyme disease

Interpretation: Suspect cases include patients who report a tick bite (or patients who report exposure to wooded/grassy areas but no known tick bite) and have symptoms consistent with Lyme disease *(fever, chills, headache, stiff neck/neck pain, arthralgia, myalgia, swollen lymph nodes, radiculopathy, paresthesia, or problems with cognition or memory). These patients do not have laboratory-confirmed Lyme disease (either no labs were performed, or they have negative results). These patients also do not have a rash consistent with erythema migrans and do not have late manifestations of disease.*

Does the case meet the suspect Lyme disease criteria? Yes/No/Uncertain

***Laboratory-confirmed Lyme disease**

Positive Lyme cultures, PCR tests, or two-tiered tests indicate laboratory-confirmed Lyme disease.

Positive two-tiered tests:

1. If the patient experienced signs or symptoms for $\leq$ 30 days before a positive or equivocal EIA or IFA, they must have a positive IgG or IgM Western Blot to have a positive two-tiered test.
2. If the patient has experienced signs or symptoms for > 30 days before a positive or equivocal EIA or IFA, they must have a positive IgG Western Blot to have a positive

two-tiered test. A positive IgM Western Blot does not confirm Lyme disease in this scenario.

** **Late manifestations of Lyme disease**

1. Musculoskeletal involvement is defined as inflammatory arthritis or recurrent and brief attacks of swelling in one or more joints that lasts for several weeks or months. Chronic progressive arthritis not preceded by recurrent, brief attacks or chronic symmetrical polyarthritis does not meet criteria for musculoskeletal involvement. Likewise, arthralgia, myalgia, or fibromyalgia syndromes alone do not meet criteria for musculoskeletal involvement.

2. Nervous system involvement is defined as one of the following:
   - Lymphocytic meningitis - inflammation of membranes covering the brain and spinal cord which can result in headache, neck stiffness, nausea, vomiting, light sensitivity or fever. A lumbar puncture is used to diagnose meningitis. Look for a laboratory test on cerebral spinal fluid with a white blood cell count ≥5 and cell count differential showing a majority of lymphocytes.
   - Cranial neuritis - particularly seventh nerve (Bell's) palsy or paralysis of facial muscles. This can result in trouble closing eyes, uneven smile, or drooling. Any of the 12 cranial nerve may be involved especially the oculomotor nerves. May be labeled as a "cranial nerve" palsy or deficit.
   - Radiculoneuropathy - inflammation of the roots of the spinal nerve which can result in numbness or tingling and increased sensitivity to pain in the arms, legs, chest, or face. Rarely causes motor weakness.
   - Encephalomyelitis - inflammation of the brain tissue which may result in sleepiness, abnormal mood swings, confusion, cognitive changes, personality or behavior changes, hallucinations, or seizures. Health care provider must explicitly use the diagnosis encephalitis or encephalomyelitis and attribute it to Lyme disease. There should be confirmatory CNS imaging with MRI or other modality.

   Headache, fatigue, paresthesia, or mildly stiff neck alone are not criteria for neurologic involvement.

3. Cardiovascular involvement is defined as acute onset of high-grade atrioventricular conduction defects that resolve in days to weeks. This may be labeled as "complete heart block" or "third degree heart block" or "high-grade AV block". Palpitations, bradycardia, bundle branch block, or myocarditis alone are not criteria for cardiovascular involvement.
